# Glomerular proteomic profiling reveals early differences between preexisting and de novo type 2 diabetes in human renal allografts

**DOI:** 10.1186/s12882-023-03294-z

**Published:** 2023-08-25

**Authors:** Anne Kipp, Hans-Peter Marti, Janka Babickova, Sigrid Nakken, Sabine Leh, Thea A. S. Halden, Trond Jenssen, Bjørn Egil Vikse, Anders Åsberg, Giulio Spagnoli, Jessica Furriol

**Affiliations:** 1 Department of Clinical Medicine, University of Bergen, Bergen, Norway; 2 Department of Medicine, Haukeland University Hospital, Bergen, Norway; 3 Institute of Molecular Biomedicine, Faculty of Medicine, Comenius University, Bratislava, Slovakia; 4 Department of Pathology, Haukeland University Hospital, Bergen, Norway; 5 Department of Transplantation Medicine, Oslo University Hospital, Rikshospitalet and University of Oslo, Oslo, Norway; 6 Metabolic and Renal Research Group, Faculty of Health Sciences UiT, The Arctic University of Norway, Tromsø, Norway; 7 Department of Medicine, Haugesund Hospital, Haugesund, Norway; 8 Department of Pharmacy, University of Oslo, Oslo, Norway; 9 Institute of Translational Pharmacology, National Research Council, Rome, Italy

**Keywords:** PTDM, T2DM, Proteomics, Tissue transplantation, Kidney

## Abstract

**Background:**

Diabetes mellitus (DM), either preexisting or developing after transplantation, remains a crucial clinical problem in kidney transplantation. To obtain insights into the molecular mechanisms underlying PTDM development and early glomerular damage before the development of histologically visible diabetic kidney disease, we comparatively analysed the proteome of histologically normal glomeruli from patients with PTDM and normoglycaemic (NG) transplant recipients. Moreover, to assess specificities inherent in PTDM, we also comparatively evaluated glomerular proteomes from transplant recipients with preexisting type 2 DM (T2DM).

**Methods:**

Protocol biopsies were obtained from adult NG, PTDM and T2DM patients one year after kidney transplantation. Biopsies were formalin-fixed and embedded in paraffin, and glomerular cross-sections were microdissected. A total of 4 NG, 7 PTDM and 6 T2DM kidney biopsies were used for the analysis. The proteome was determined by liquid chromatography-tandem mass spectrometry. Relative differences in protein abundance and significantly dysregulated pathways were analysed.

**Results:**

Proteins involved in cell adhesion, immune response, leukocyte transendothelial filtration, and cell localization and organization were less abundant in glomeruli from PTDM patients than in those from NG patients, and proteins associated with supramolecular fibre organization and protein-containing complex binding were more abundant in PTDM patients. Overall, proteins related to adherens and tight junctions and those related to the immune system, including leukocyte transendothelial migration, were more abundant in NG patients than in transplanted patients with DM, irrespective of the timing of its development. However, proteins included in cell‒cell junctions and adhesion, insulin resistance, and vesicle-mediated transport were all less abundant in PTDM patients than in T2DM patients.

**Conclusions:**

The glomerular proteome profile differentiates PTDM from NG and T2DM, suggesting specific pathogenetic mechanisms. Further studies are warranted to validate these results, potentially leading to an improved understanding of PTDM kidney transplant pathophysiology and to the identification of novel biomarkers.

**Supplementary Information:**

The online version contains supplementary material available at 10.1186/s12882-023-03294-z.

## Background

Diabetes mellitus (DM) is a major cause of end-stage renal disease (ESRD) [1]. Treatment options for patients with ESRD include dialysis and transplantation, the latter being superior in terms of patient survival, quality of life, and economic impact [2].

Chronic kidney disease (CKD) affects 20–40% of patients with DM [3]. Although its pathophysiology is not fully understood, diabetic kidney disease (DKD) is thought to originate from a glucose-driven increase in glomerular filtration and tubular reabsorption leading to an overload of nephron components and their subsequent injury. Posttransplantation diabetes mellitus (PTDM) is a common complication in renal transplant recipients that promotes the subsequent development of other diseases, such as cardiovascular disorders and CKD. Risk factors for PTDM include the use of immunosuppressive drugs, posttransplant viral infections and genetic predispositions of the kidney recipient [4], in addition to the commonly known risk factors for type 2 diabetes (T2DM) [5]. Approximately 60% of nondiabetic patients present hyperglycaemia in the immediate posttransplant phase [6], and 16% to 37% will develop PTDM [7, 8].

Typical histopathological findings in DKD include thickening of the glomerular basement membrane, mesangial matrix expansion, nodular glomerulosclerosis, and arteriolar hyalinosis [9]. DM developing after transplantation displays similar features but is also frequently associated with allograft rejection-induced tubulointerstitial and vascular alterations, as well as histological features related to viral infection or immunosuppressive drug-related toxicity [10].

DKD is similarly detectable in patients with recurrent T2DM and PTDM, with an incidence of 25% and 30%, respectively, within 6 years following transplantation [11]. However, the emergence of specific additional diabetic complications, occurring after ~ 1.8 years, is accelerated in PTDM patients compared to nontransplanted T2DM patients [12].

These differences suggest that at least partially dissimilar mechanisms might be involved in the pathophysiology of recurrent kidney injury in T2DM patients following transplantation and in PTDM patients. Indeed, PTDM and its complications are increasingly recognized as a unique form of diabetes, and evidence-based treatment regimens currently used in patients with T2DM are not directly transferable to patients with PTDM [13, 14].

To date, proteomic studies have mainly been performed on blood and/or urine samples [15]. However, tissue biopsies, while more difficult to obtain, might provide valuable data that improves the understanding of specific tissue characteristics in normal and pathological states [16].

In this pilot study, we isolated glomeruli from formalin-fixed paraffin-embedded (FFPE) kidney biopsies by laser caption microdissection (LCM) and performed proteomic analysis. By comparing the proteomes of histologically normal glomeruli from normoglycaemic (NG), T2DM, and PTDM patients one year after kidney transplantation, we sought to gain new insights into the molecular mechanisms underlying PTDM development and early glomerular damage prior to the development of histologically visible diabetic kidney disease. Similarities and differences detectable in NG, PTDM and T2DM glomerular protein profiles could also be addressed by data integration, network analysis and immunohistochemistry.

To the best of our knowledge, this is the first time that proteomics from microdissected glomeruli has been investigated in renal allografts of patients with DM.

## Methods

### Study design and patients

Adult renal transplant recipients underwent an in-depth investigation, including protocol biopsies, an oral glucose tolerance test (OGTT), and a HbA1C test, 8–10 weeks and one year after transplantation at Rikshopitalet, Oslo University Hospital, Oslo, Norway. Kidney biopsy samples were collected between 2014 and 2017. Data were stored in a local registry, and biopsies were stored in a diagnostic biobank. Patients signed a written informed consent form. The study was approved by the regional ethics committee of the South-Eastern Norway Regional Health Authority (REK sør-øst: 2016/912).

Adult patients with a valid glucose metabolism status and a protocol biopsy at 1 year after transplantation, stable renal function with < 20% deviation in serum creatinine within the last two months, and immunosuppressive therapy stable for more than three months before protocol biopsy at 1-year examination were selected. Immunosuppressive treatment was similar in all groups (Table 1). Insulin and other antidiabetics were administered to patients with PTDM and T2DM.

**Table 1:** Summary of patients´ treatment

	**PTDM**	**T2DM**	**NG**
**Corticosteroids**	7/7 (100%)	6/6 (100%)	4/4 (100%)
**Mycophenolate**	7/7 (100%)	6/6 (100%)	4/4 (100%)
**Tacrolimus**	7/7 (100%)	6/6 (100%)	4/4 (100%)
**Insulin**	0/8 (0%)	6/6 (100%)	0/4 (0%)
**Other antidiabetics**	3/7 (43%)	1/6 (16,6%)	0/4 (0%)
**Antihypertensive drugs**	6/7 (86%)	6/6 (100%)	4/4 (100%)

Exclusion criteria included an estimated glomerular filtration rate (eGFR) < 30 mL/min/1.73 m^2^ and any clinical and/or histological manifestations of graft rejection. Ultrasound-guided renal biopsies were obtained using an 18G needle. Tissues were formalin-fixed and paraffin-embedded (FFPE) for conservation and further analyses.

Three different groups of patients were analysed: 1) patients with PTDM (*n* = 8), 2) patients with T2DM (*n* = 8), and 3) patients with NG (*n* = 8). To exclude confounding pathologies, glomeruli that fulfilled one or more of the following criteria were excluded from further analyses: global sclerosis, ischaemia, and periglomerular inflammation. In the case of segmental sclerosis, only sections that appeared to be healthy were microdissected. Interstitial fibrosis and tubular atrophy in allograft biopsies were classified using the Banff classification [17]. All samples showed less than 25% interstitial fibrosis and/or tubular atrophy. The total number of glomeruli per sample and the percentages of sclerotic glomeruli and glomeruli with glomerulonephritis, interstitial fibrosis and tubular atrophy are described in Table S1.

### Sample preparation and laser capture microdissection

Ten-micrometre-thick FFPE sections were deparaffinized, rehydrated, stained, and scanned with ScanScope XT Aperio. Selected FFPE sections were mounted on preirradiated polyethylene naphthalate slides (MembraneSlide 1.0 PEN, Carl Zeiss MicroImaging GmbH), and a total area of approximately 2 million μm2 dissected glomeruli for each sample tissue was isolated using a PALM Microbeam System (P.A.L. M, Bernried, Germany) and pressure catapulted into a tube cap (AdhesiveCap 500 clear, Zeiss). Microdissected FFPE glomeruli were stored at − 20 °C until peptide extraction. Then, they were resuspended in 10 μl of lysis buffer (0.1 M Tris pH 8, 0.1 M dithiothreitol [DTT], 4% sodium dodecyl sulfate). A filter-aided sample preparation (FASP) protocol based on trypsin digestion was used to extract the proteins [18]. Digested peptides were eluted and desalted using Oasis HLB µElution plates (Waters, Milford, MA), dried by a vacuum centrifuge, and rehydrated in 2% acetonitrile (ACN) and 0.1% formic acid (FA). NanoLC-ESI-LTQ Orbitrap Elite was used for tandem mass spectrometry.

### Immunohistochemistry

Antibodies against the adhesion-related proteins MLLT4 (RRID:AB_10599291) and CTNND1 (RRID:AB_1846068) and the enzyme LHPP (RRID:AB_1079250) from Atlas Antibodies (Sigma‒Aldrich) were selected for immunohistochemical verification. These antibodies have been validated as described in the Human Protein Atlas (https://www.proteinatlas.org/). Immunohistochemistry was performed in accordance with the manufacturers’ instructions (Table S2). All immunoreactions were visualized using 3,3'-diaminobenzidine (DAB, Dako), counterstained with haematoxylin (Dako), dehydrated, and placed under a cover-slip using a nonaqueous mounting medium.

Stained slides were scanned in a ScanScope™ system (Aperio, Vista, California, USA) at the Department of Pathology at Haukeland University Hospital in Bergen, Norway. The generated digital slides were viewed in an Imagescope 12 (Leica Biosystems, Nussloch, Germany). Glomeruli were annotated in each slide, and quantification of IHC staining was carried out using the colour deconvolution algorithm version 9.1 (Aperio) after adjusting for the default parameters for each staining. Visualization data was obtained by dividing the number of strong positives by the total number of pixels. Data are presented as box plot graphs, and the Mann‒Whitney test was used to assess statistical significance. A p value ≤ 0.05 was considered statistically significant. Graphs and statistics were generated using SPSS Statistics 27 (IBM).

### Statistics and computational analysis

Raw mass spectrometer files were analysed using MaxQuant v 1.6.1.0 [19]. MS/MS spectra were searched in the Andromeda search engine against the forwards and reverse Human UniProt database (Swissprot reviewed canonical and isoforms 23.04.18). Label-free quantification was used to identify the relative amount of proteins in each sample. Proteome analysis was performed using Perseus (v. 1.5.5.3, RRID:SCR_015753). Briefly, data were filtered and transformed (log2 (x)). Rows with < 70% valid values in at least one group were excluded. Imputation of missing data was performed by random numbers drawn from a normal distribution with a width of 0.3 and downshift of 1.8 applied to each expression column separately, and data were normalized using Z score. Data are available via ProteomeXchange with identifier PXD042188 [20].

SPSS (IBM SPSS Statistics v.25; RRID:SCR_019096) was used for general statistics. Proteins were compared to the complete human proteome to determine overrepresented Gene Ontology (GO) categories. The enrichment analysis was performed using STRING-db (v. 11.5) [21, 22]. GO, the ShinyGO v. 0.76 (http://bioinformatics.sdstate.edu/go/), [23, 24] and Kyoto Encyclopedia of Genes and Genomes (KEGG) [25] enrichment analyses were used for pathway analysis.

The t test was used for data comparisons, and *p* values ≤ 0.05 were considered statistically significant.

## Results

### Sample selection and analysis

Proteomic analysis was performed on glomeruli isolated from FFPE kidney biopsies sampled one year after kidney transplantation. Three groups of adult patients were studied: NG patients (*n *= 8), patients with PTDM (*n* = 8), and patients with pretransplantation T2DM (*n* = 8). Following proteomic analysis, two samples from the T2DM group, one from the PTDM group and four from the NG group were excluded because the number of proteins identified was substantially lower (< 300) than that from the other samples, or due to paucity of biopsy material and/or not normally distributed intensity relative to the base peak. Therefore, the final number of samples included in the analysis was *n* = 4 for NG, *n* = 6 for T2DM and *n* = 7 for PTDM. The clinical characteristics of the three groups of patients included in the final analyses are summarized in Table 2. Patients did not present graft dysfunction or micro- or macrovascular complications at the time of the biopsy. While T2DM patients were diagnosed with DM between 2 and 31 years before the surgery (average: 16.8 years), in the PTDM subgroup, DM was diagnosed within the first 8 weeks after transplantation in 4 patients and between 8 weeks and one year in 3. For NG patients, follow-up 4–5 years after biopsy indicated that none of them had developed DM. PTDM and NG patients did not present proteinuria at the time of biopsy, whereas this was present in 2 T2DM patients.

**Table 2:** Clinical characteristics of the final cohort

	**PTDM (*n* = 7)**	**T2DM (*n* = 6)**	**NG (*n* = 4)**	***p*-value** **PTDM vs T2DM**	***p*-value** **PTDM vs NG**	***p*-value** **T2DM vs NG**
Sex, Male/Female	4/3	5/1	2/2			
Age (years), mean (± SD)	60,9 (12.3)	64.3 (12.2)	53 (5.2)	0.620	0.176	0.083
Range	46–76	52–81	48–58			
BMI (kg/m^2^), mean (± SD)	27.2 (1.6)	30.0 (1.5)	26.2 (2.2)	0.007	0.413	0.010
Weight gain post-tx (Kg), mean (± SD)	-2.7 (5.7)	2.2 (4.6)	0.25 (0.5)			
Fasting glucose-pre-tx (mmol/L), mean (± SD)	5.3 (0.7)	11.1 (5.7)	5.6 (0.3)	0.051	0.277	0.065
Range	4.8–6.3	4.9–20.7	5.4–5.9			
HbA1C test, %, mean (± SD) pre-tx	5.2 (0.4)	6.5 (0.7)	5.3 (0.2)	0.001	0.866	0.006
Range	4.5–5.7	5.8–7.5	5.0–5.5			
HbA1C test, %, mean (± SD) post-tx	6.7 (0.8)	7.4 (0.8)	5.2 (0.2)	0.123	0.008	< 0.001
Range	5.6–8.0	6.5–8.6	5.0–5.5			
eGFR (mL/min/1.73 m^2^), mean (± SD) post-tx	53 (11)	39 (8)	65 (11)	0.026	0.121	0.003
Hypertension post-tx %	71	100	75			
Donor (DD/LD)	3/4	5/1	3/1			
Donor age (years), mean (± SD)	47.8 (15.8)	64.2 (15.4)	57.0 (14)	0.076	0.346	0.477

*Abbreviations*: *BMI* Body mass index, *pre-tx* Pre-treatment, *HbA1C* Glycated hemoglobin, *post-tx* Post-treatment, *DD* Dead donor, *LD* Living donor

### Glomerular proteomic profiling and identification of differentially abundant proteins

A total of 1329 proteins were detected in the glomerular tissue samples. Of these proteins, 1237 could be identified by at least one unique peptide sequence and were used for further analyses. A full list of the identified proteins is provided in Supplementary Table 3.

**Fig. 1:**
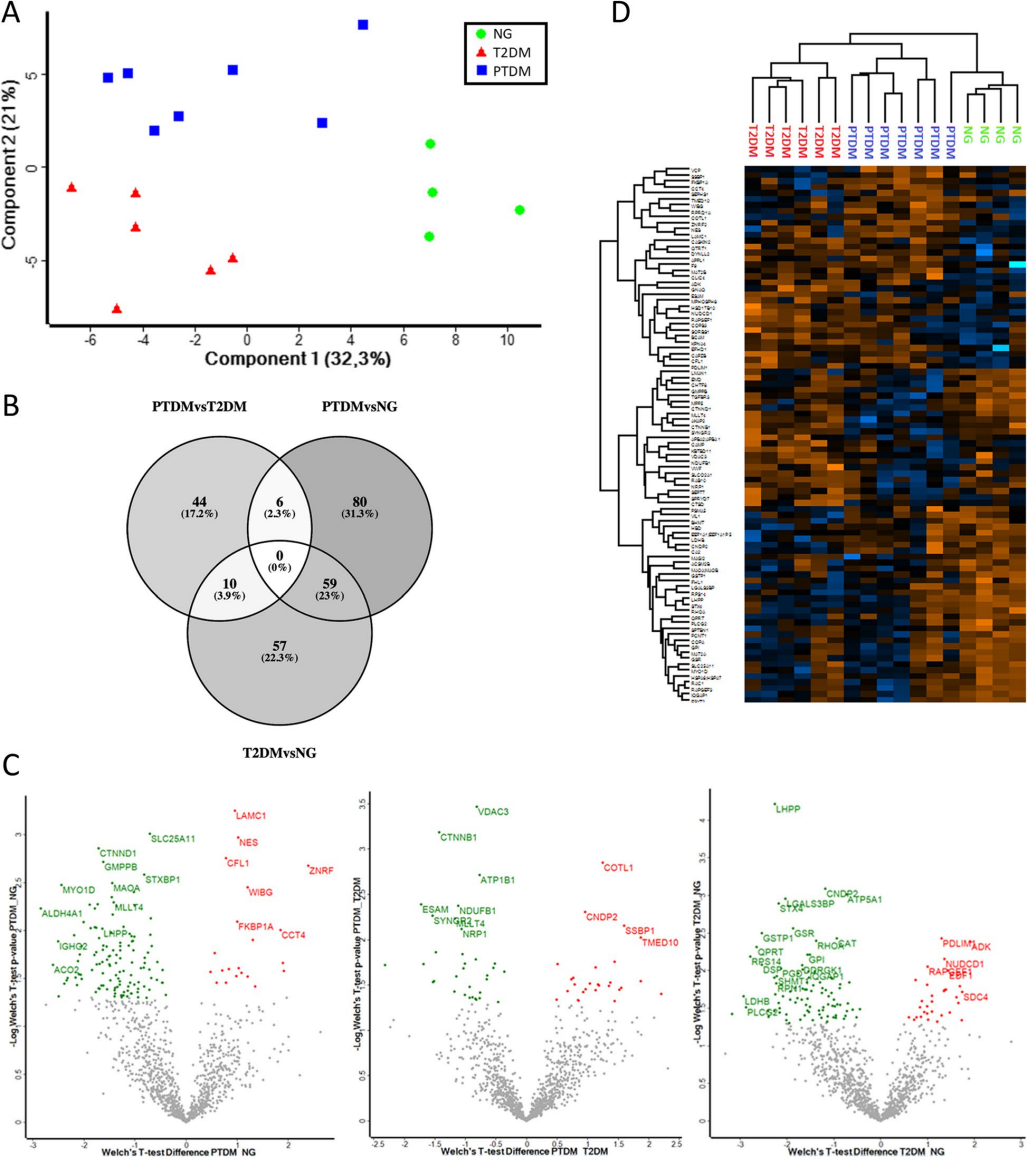
NG, PTDM, and T2DM protein profile analysis. **A** Protein Principal component analysis (PCA) based on protein data from PTDM (blue dots), T2DM (red dots), and NG (green dots). Group NG is separated along with principal component 1 (PC1) from both PTDM and T2DM, whereas PTDM and T2DM are separated along with component 2 (PC2). **B** Venn diagram depicting the overlap of proteins differentially expressed in the three statistical comparisons obtained using http://bioinfogp.cnb.csic.es/tools/venny. **C** Proteins up or down represented in each comparison with a selection of protein names represented (**D**) Hierarchical clustering of proteins differentially expressed in glomeruli from patients with PTDM, T2DM and NG (average linkage, Pearsons correlation, Z-score)

The molecular mass of the detected proteins ranged between 5 and 670 kDa. Initial exploratory assessment of the dataset was performed using a two-dimensional principal component analysis (PCA) based on ANOVA significantly differentially regulated proteins (*n* = 90). A clear separation between the three groups under investigation was evident when the samples were plotted on these two axes, with PTDM samples located slightly closer to the NG group than the T2DM samples (Fig. 1A). These data suggest that variations in the glomerular proteomes may allow discrimination among these three different groups.

An initial proteome analysis showed that 144, 126 and 60 glomerular proteins were differentially abundant in NG compared to PTDM, in NG compared to T2DM, and in PTDM compared to T2DM, respectively. The numbers of shared differentially abundant proteins are summarized in Fig. 1B and Table S4. Volcano plots comparatively depicting the differential abundance of specific proteins in defined glomerular specimens are presented in Fig. 1C. A full list of proteins significantly differentially abundant in each group is provided in Table S3.

Hierarchical clustering of proteins differentially abundant in the PTDM, T2DM and NG groups was performed to identify specific expression patterns. Notably, T2DM, NG and, to a somewhat lower extent, PTDM samples clustered with a clear differentiation pattern (Fig. 1D).

### Differentially abundant proteins in NG and PTDM glomeruli

The characteristic feature of patients in the PTDM group was that they had developed DM following transplantation, whereas patients in the NG group had not, which may indicate renal transplant-specific effects, including immunosuppressive treatment. To obtain insights into involved pathways and potential early disease markers, we performed category enrichment analysis of proteins differentially abundant in NG and PTDM glomeruli.

**Fig. 2:**
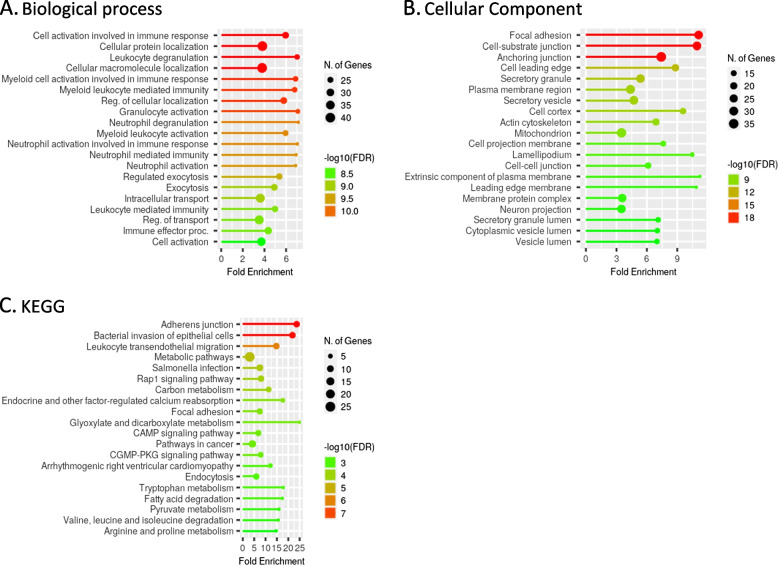
Gene ontology (GO) and KEGG pathway enrichment analysis of proteins underrepresented in PTDM vs. NG. **A** GO Biological Process; **B** Cellular component; **C** KEGG. The 20 most enriched pathways are represented. (http://bioinformatics.sdstate.edu/go/)

A large majority of differentially abundant proteins (123 of 144) were less abundant in PTDM glomeruli than in NG glomeruli (PPI enrichment *p* value < 1.0e-16). In particular, proteins related to adhesion, including the nephrin-family proteins SPATN1, SPTBN1, MAGI2, IQGAP1 and KIRREL, the immune system, leukocyte transendothelial filtration, and cell localization and organization were downregulated in glomeruli from PTDM patients compared to NG patients (Fig. 2A, B, C).

The 21 less abundant proteins in NG compared to PTDM glomeruli also had significant PPI enrichment (*p* = 0.000765) and were mainly associated with supramolecular fibre organization (enrichment FDR: 6.3 × 10^–5^; fold enrichment: 10.6) and protein-containing complex binding (enrichment FDR: 1.6 × 10^–6^; fold enrichment: 8.3). In addition, tacrolimus binding protein FKBP1A was significantly overrepresented in glomeruli from PTDM patients compared to NG patients. The results for NG compared to T2DM were largely similar to those for NG compared to PTDM (Table S3) glomeruli. However, the expression levels of tacrolimus binding protein FKBP1A in NG and T2DM glomeruli were similar (*p* = 0.72).

### Nondiabetic and diabetic glomerular proteomes in kidney transplant patients: gene ontology and protein interaction analysis

Data generated in our study allowed proteomic profiling of glomeruli from transplanted patients with DM, irrespective of its preexistence or “de novo” posttransplant development. Therefore, we sought to identify proteins differentially abundant between NG glomeruli and the combination of PTDM and T2DM glomeruli. A category enrichment analysis was performed and revealed that, overall, these proteins were biologically connected as a group (PPI enrichment *p* = 7.9 × 10^–7^).

**Fig. 3:**
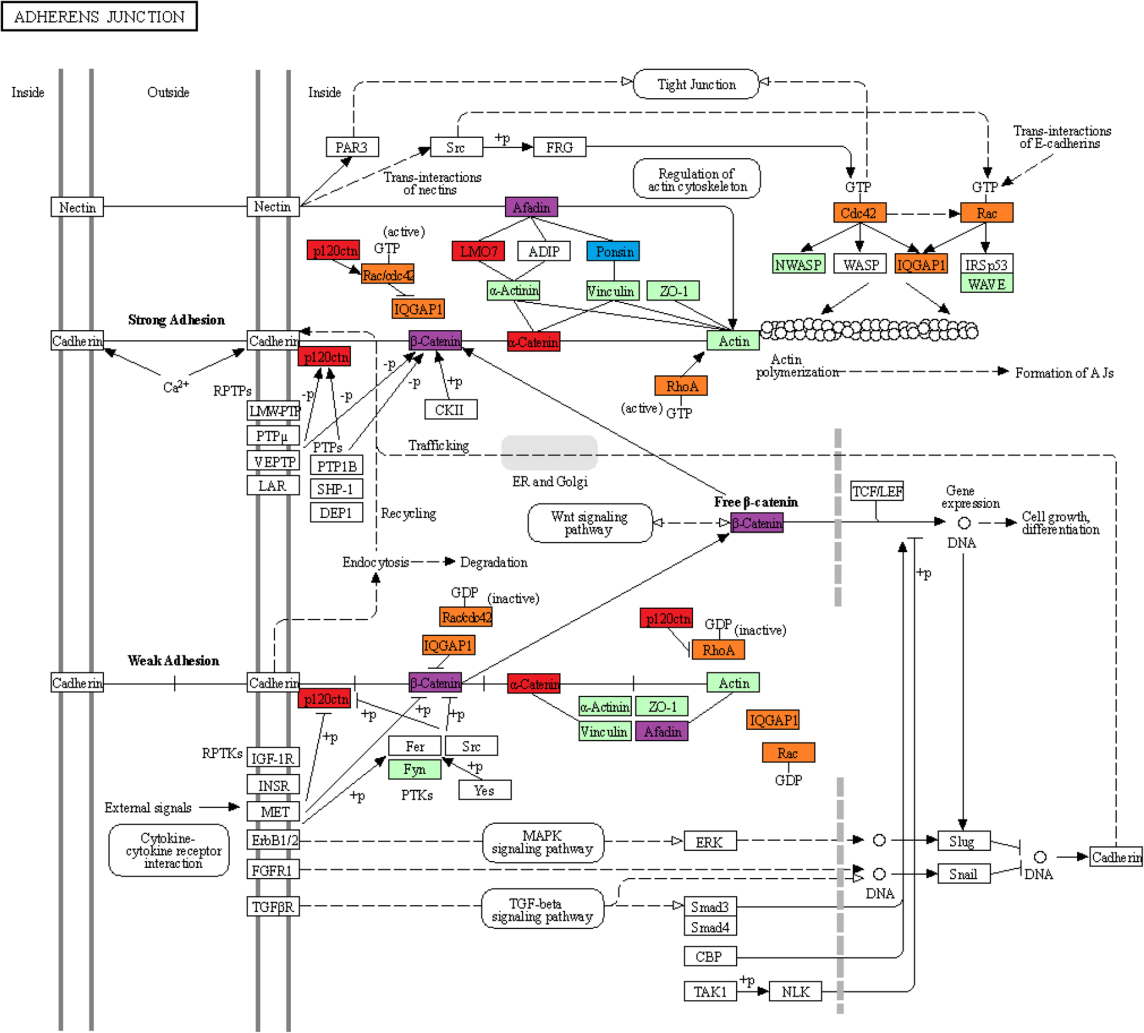
Map of the KEGG pathways “Adherens junction”. Violet: proteins underrepresented in PTDM compared to both, NG and T2DM; Blue: proteins underrepresented in PTDM compared T2DM; Red: proteins underrepresented in PTDM compared to NG; Orange: proteins highly represented in NG compared to both, PTDM and T2DM; Green: proteins detected by MS in our dataset but not significantly differentially abundant in any comparison. Modified from Kyoto Encyclopedia of Genes and Genomes (KEGG)

**Fig. 4:**
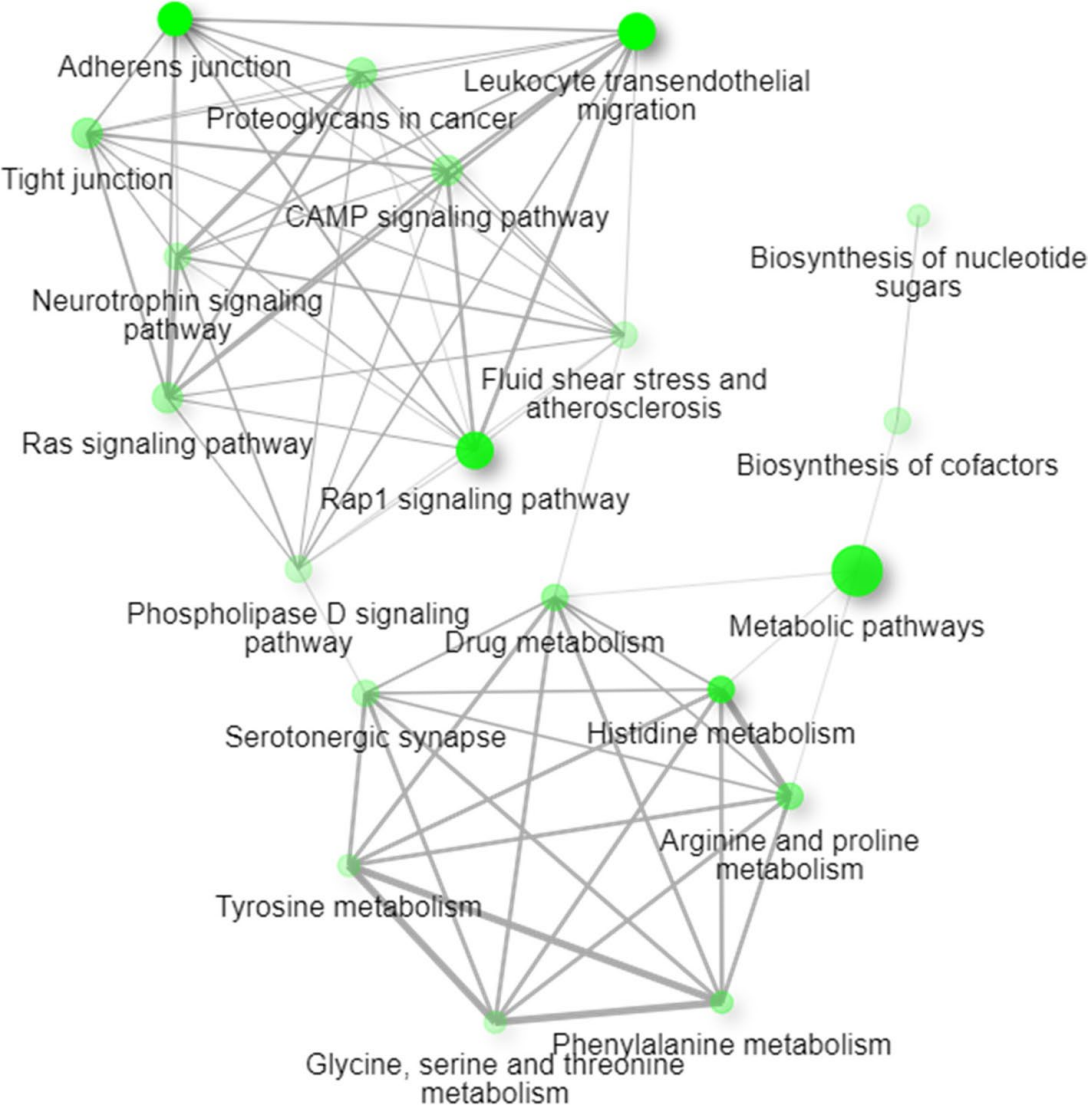
Network representation using KEGG terms of differentially abundant proteins overrepresented in NG compared to PTDM and T2DM. The 20 most enriched pathways are represented. (http://bioinformatics.sdstate.edu/go/)

In particular, a number of proteins were overrepresented in glomeruli from NG patients compared to all DM patients (PPI enrichment *p* = 1.27 × 10^–5^). Notably, proteins included in biological processes related to cell‒cell communication, such as adherens junctions (Fig. 3) and tight junctions, and the immune system, such as leukocyte transendothelial migration, were more abundant in NG samples than in all DM samples, irrespective of their PTDM and T2DM nature (Fig. 4). In contrast, in the group of proteins less abundant in NG, no significant PPI enrichment was observed, likely due to the low number of proteins included (*n* = 13).

Taken together, these results reveal alterations in transport regulation, cell organization and communications, and in the immune system, as detected in glomeruli from all transplanted patients with DM, irrespective of the timing of its development.

### The proteomes of the two diabetic groups: gene ontology and protein interaction analysis

PTDM is characterized by “de novo” development following transplantation. Although our data consistently documented commonalities between PTDM and T2DM glomerular proteomic profiles, we addressed the identification of the few differentially expressed proteins, potentially suggesting specificities of PTDM development.

Indeed, proteins involved in insulin secretion, such as VAMP2, GNAQ and ATP1B1, and cell‒cell junctions and adhesion, including RAB10, ESAM, ponsin (SORBS1), afadin (MLLT4), and catenin beta (CTNNB1), appeared to be downregulated in PTDM compared to T2DM. Von Willebrand factor (VWF), whose circulating levels were previously reported to be increased in patients with CKD and ESRD compared with healthy control individuals, was also overrepresented in T2DM glomeruli [26, 27]. Furthermore, VAMP2, which belongs to the SNAP receptor protein family (SNARE) and has been associated with insulin resistance in T2DM, was underrepresented in PTDM [28, 29]. Interestingly, the expression level of the tacrolimus binding protein FKBP1A was also slightly, although not significantly, lower in PTDM (*p* = 0.06).

**Fig. 5:**
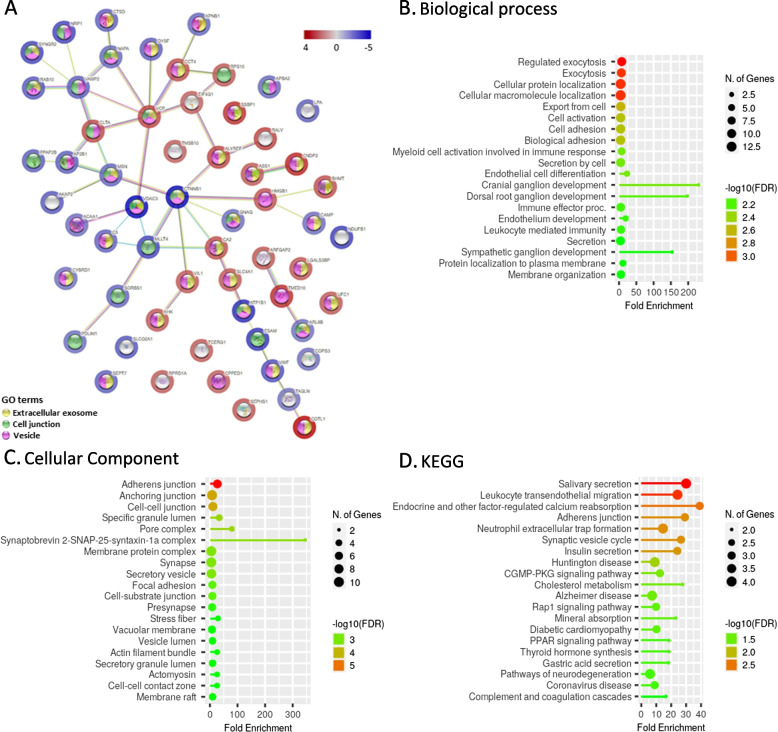
PTDM and T2DM protein interaction and pathway enrichment analyses. **A** Protein–protein Interaction network using proteins with significantly different abundance in PTDM and T2DM. The network nodes represent specific proteins. Blue halo refers to protein abundance lower in PTDM vs. T2DM; red halo refers to higher abundance in PTDM vs. T2DM. Edges represent protein–protein associations. (http://www.string-db.org). **B** GO Biological Process pathway enrichment analysis of proteins underrepresented in PTDM vs. T2DM. **C** Cellular component pathway enrichment analysis of proteins underrepresented in PTDM vs. T2DM. **D** KEGG pathway enrichment analysis of proteins underrepresented in PTDM vs. T2DM. The 20 most enriched pathways are represented (http://bioinformatics.sdstate.edu/go/)

In contrast, proteins overabundant in glomeruli from PTDM patients compared to those from T2DM patients included CCT4, which is a component of the T-complex protein ring that has been proposed as a biomarker of glomerular hyperfiltration [30, 31], and CNDP2, which is possibly also associated with diabetic kidney disease [32] (Fig. 5A).

In the total analysis of differentially abundant proteins, we found a protein‒protein interaction (PPI) enrichment *p* = 0.0118. Thus, the PPI network contained more interactions than expected in a set of similar size, and the proteins detected could be considered at least partially biologically connected as a group.

GO analysis, focusing on significantly enriched categories with an FDR < 0.05, indicated that proteins related to exocytosis and vesicle lumen and adherens junctions were overrepresented in T2DM (FDR ≤ 10^–4^, data not shown) (Fig. 5B, C and D).

Taken together, these results show that proteomic alterations of cell‒cell and cell-extracellular matrix structures in PTDM occur early but remain undetectable at the histological level since the glomeruli selected for microdissection looked normal under the microscope.

### Immunohistochemical analysis for the validation of differentially abundant proteins

To validate the altered pattern of protein abundance observed by proteomic evaluation, MLLT4, CTNND1, and LPHH proteins were selected for immunohistochemical (IHC) analysis based on their high degree of dysregulation between the groups under investigation and the availability of Prestige Antibodies (Sigma‒Aldrich), as described in the Human Protein Atlas (https://www.proteinatlas.org/). MLLT4 and CTNND1 are adherens junction-related proteins that are also linked to leukocyte transendothelial migration (TEM) and adhesion. In the proteomics data, MLLT4 levels were lower in the PTDM group than in both the T2DM and NG groups, and CTNND1 levels were significantly lower in the group PTDM than in the NG group. Additionally, a similar trend was observed between the T2DM and NG groups, and this trend was consistent with the disruption in adherens junctions in PTDM and T2DM that was more apparent in PTDM. LHPP, a histidine phosphatase that has been proposed as a proliferation marker [33, 34], was expressed to lower extents in the PTDM and T2DM groups compared to the NG group.

**Fig. 6:**
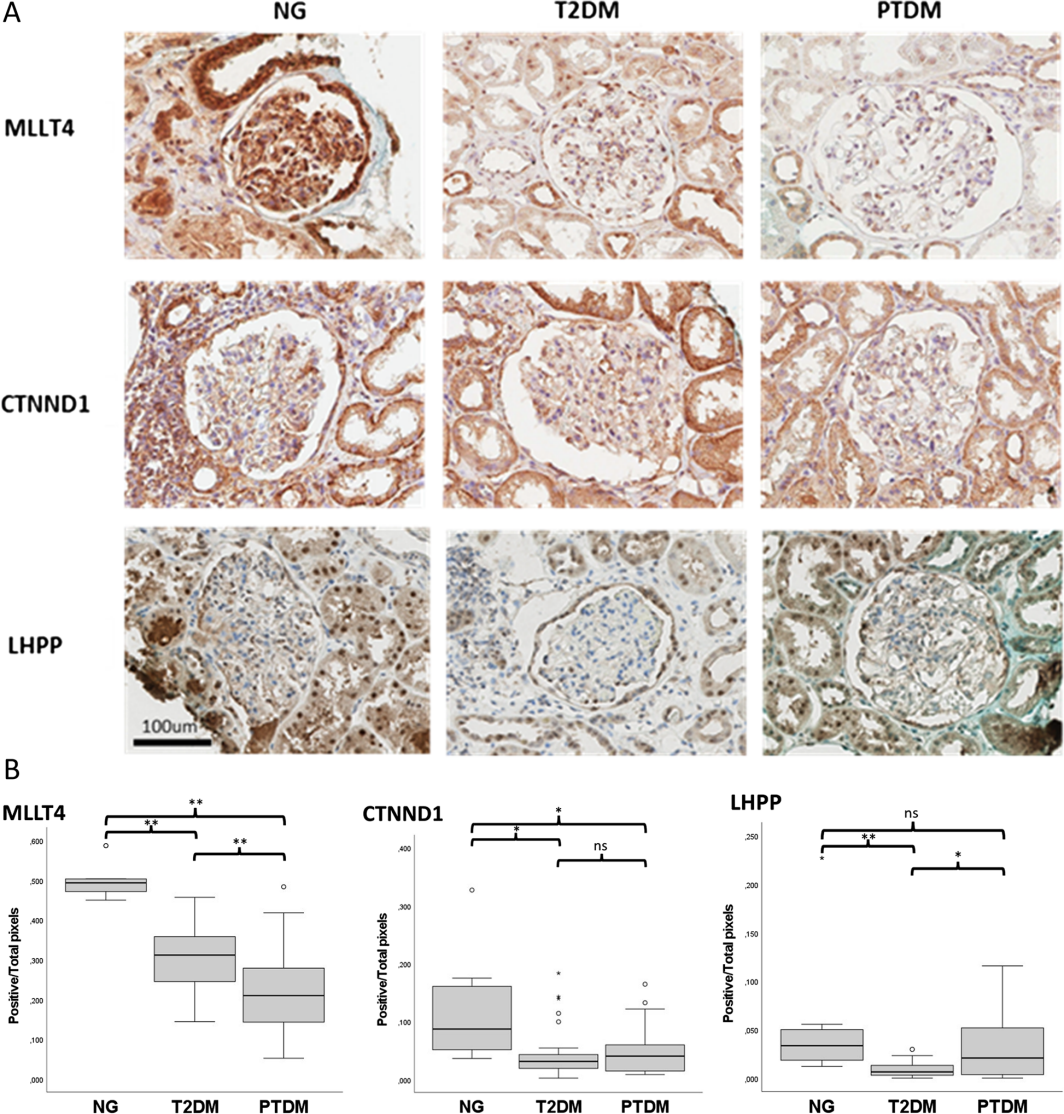
Immunohistochemical analysis of proteins of differential abundance as detected by proteomic profiling. **A** Representative IHC detection of MLLT4, CTNDD1 and LHPP in NG, T2DM and PTDM glomeruli (**B**) Boxplot representing pixel analysis-based protein quantification for MLLT4, CTNND1 and LHPP in NG, T2DM and PTDM. FFPE from six different biopsies from each group were stained with each specific antibody. Statistical analysis was performed using the Mann–Whitney test. *p-value ≤ 0.05; **p-value ≤ 0.005

The immunohistochemistry results showed changes consistent with the quantitative proteomics results, as depicted in Figs. 6A and B. Pixel analysis showed that the expression levels of MLLT4 and CTNND1 were significantly higher in NG samples than in PTDM samples, and MLTT4, CTNND1 and LHPP expression levels were lower in T2DM samples than in NG samples. Moreover, the expression levels of MLLT4 were significantly lower in PTDM samples than in T2DM samples.

## Discussion

PTDM is a frequent condition following kidney transplantation and has an adverse impact on clinical outcome and patient survival. Although previous literature indicates that PTDM is a variant of T2DM that might need different therapeutic interventions [35, 36], little is known about the molecular mechanisms underlying this disease. To gain new insights into DM development and early glomerular damage in PTDM compared to T2DM following transplantation, we combined proteomics on glomeruli from kidney transplant biopsies with data integration and network analysis. In this study, we focused on glomeruli, as podocyte dysfunction and glomerular enlargement have been described as key characteristics of DKD [37, 38]. Growing evidence implicates inflammatory cells modulating local immune responses in the pathogenesis of diabetic kidney disease [39]. Indeed, increased levels of pro-inflammatory cytokines and chemokines and an activation state of lymphocytes and myeloid cell populations have been described in T2DM [39, 40]. Furthermore, persistent complement protein activation has not only been associated with insulin resistance and complications in DM [41, 42] but also with diabetic nephropathy [43]. Thus, it has been proposed as a therapeutic target in diabetic kidney disease [44]. These features were also found to be associated with increased apoptosis of adipocytes and islet cells and tissue fibrosis. Moreover, the link between obesity and inflammation is also well established [45].

Nevertheless, surprisingly, our data indicate that proteins associated with immune responses are underrepresented in glomeruli from transplanted patients with PTDM or T2DM compared to NG patients, even if immunosuppressive treatment was similar. However, the expression of complement proteins C3, C1QS, C1S, C4, and C8B was not significantly different between groups. However, C5 appeared to be lower in PTDM than in T2DM, and C6 was higher in T2DM than in NG. 

Active cell‒cell communication, achieved through direct contact or via secreted factors, is required to maintain homeostasis in all multicellular organisms. Interestingly, proteins driving cell‒cell communication were underrepresented in both the PTDM and T2DM groups compared to the NG group.

These underrepresented proteins included the nephrin family interaction-related proteins SPTAN1, SPTBN1, IQGAP1, KIRREL, and MAGI2 and the transmembrane 4 superfamily member CD151. CD151 is essential for the proper assembly of glomerular and tubular basement membranes in the kidney [46]. Moreover, nephrin family interaction-related proteins are closely associated with podocytes and kidney filtration, as they all function as scaffolds connecting junctional membrane proteins to the cytoskeleton in the nephrin–slit diaphragm protein complex, which is essential for glomerular ultrafiltration. However, in nephrotic syndrome, podocyte architecture is lost, with foot process effacement and loss of slit diaphragms, leading to proteinuria [47, 48]. Our finding of the downregulation of the nephrin family interaction-related proteins are in accordance with the alterations observed in podocytes and glomeruli during DKD development [38]. Intriguingly, in a study on microdissected glomeruli from kidney autopsies of diabetes patients with nephropathy (DN) and patients without diabetes or renal disease (ND), nephronectin, a protein also related to cell‒cell adhesion, was found to be overexpressed in DN. Although we did not find any difference in nephronectin, probably due to the remodelling of the extracellular matrix occurring in transplantation [49], we found that other proteins, such as clusterin, laminin gamma and collagen, Type VI, alpha, were significantly modified in DN. These findings were similar to our current findings [50]. Clusterin was especially interesting, as it has been proposed as a biomarker of nephrotoxicity [51]. Moreover, in our study, it was also significantly overexpressed in PTDM and T2DM compared to NG.

Vesicle-mediated transport of proteins, lipids, nucleic acids and other molecules delivers information within and between cells. Abnormal extracellular vesicles can contribute to the occurrence of and complications associated with diabetes by inducing insulin resistance [52]. Accordingly, extracellular vesicles from the urine and circulation have gained significant interest as potential diagnostic biomarkers in renal diseases [53] and DM [54]. The STX4 protein facilitates the fusion of glucose transporter 4 (GLUT4) vesicles with the plasma membrane, thereby eliminating glucose from the circulation [55, 56]. In our study, STX4 was expressed to lower extents in T2DM and PTDM than in NG. Thus, differences in extracellular vesicle-related protein profiles could provide therapeutic targets for the treatment and prevention of kidney disease in posttransplant patients.

Interestingly, while cell‒cell communication, including leukocyte transendothelial migration, appeared to be disrupted in glomeruli from both PTDM and T2DM patients compared to NG patients, these alterations were particularly noticeable in PTDM patients. Indeed, the adherens junction-related proteins CTNNB1, MLLT4 and SORBS1 were less abundant in PTDM than in T2DM, although cadherin and nectin were undetectable. The latter is probably because our water-based protein extraction method fails to isolate lipophilic membrane proteins [57, 58]. Leukocyte transendothelial migration is a multistep process that begins with adhesion. This is followed by firm adhesion and ends with either transcellular or paracellular passage of the leukocyte across the endothelial monolayer [59]. Different types of activated leukocytes play crucial roles in the pathogenesis of kidney diseases. Although there is growing evidence for inflammatory cells that modulate the local response and thus increase inflammation in diabetic kidneys, the precise mechanisms are still unclear [40].

The differences in inflammation between PTDM and T2DM could be influenced by BMI as well as long-term DM. However, although the BMI of PTDM patients was not significantly different from that of NG patients, proteins related to transendothelial migration were still expressed to lower extents in PTDM. Overall, these data suggest that despite similar immunosuppressive treatment, proteins associated with leukocyte transendothelial migration are overrepresented in the NG group compared to both PTDM and T2DM patients.

However, notably, diabetic kidney disease markers, such as CCT4 and CNDP2, were more abundant in glomeruli from PTDM patients than in those from T2DM patients.

Limitations of our work should be acknowledged.

In particular, while age and kidney donor age did not significantly differ in PTDM and T2DM patients, higher body mass index and lower eGFR in T2DM patients could have played a role in the elicitation of the observed differential protein profiles. Additionally, all the patients in this study were treated with tacrolimus, a drug associated with a higher incidence of diabetes mellitus after renal transplantation [60], and corticosteroids, promoting increased blood glucose levels and insulin resistance [5]. Moreover, the number of patients in each group is relatively low, and confounding factors, such as differences in medication, cannot be dismissed.

Nevertheless, our results suggest that cell‒cell communication and organization are decreased in PTDM compared to T2DM and NG. This finding is consistent with a loss of glomerular structure and a faster progression of DKD in these patients despite the apparently more favourable clinical factors in PTDM compared to T2DM. It is also interesting to note that the cell adhesion and metabolism-related molecular pathways were also similarly disrupted in both PTDM and T2DM compared to NG.

Thus, despite patient heterogeneity and the limited statistical power of our study, proteome quantitation appears to be able to differentiate the three posttransplantation groups of patients by PCA and hierarchical clustering analysis.

These results can be considered a first approach to improve our understanding of the pathogenesis of glomerular filtration barrier alterations in transplantation and DM development. Future studies are warranted to address the reproducibility of these results in other cohorts.

## Conclusions

Proteomics studies in kidney disease have mainly been performed using blood and/or urine samples [15] due to the difficultly in accessing posttransplant tissue biopsies. However, these specimens, while more difficult to obtain, might provide valuable data to clarify specific tissue characteristics in normal and pathological states [16]. This is the first pilot study to perform proteomics analysis of microdissected glomeruli from posttransplant PTDM, T2DM, and NG patients.

By revealing differential molecular profiles in glomeruli from PTDM, T2DM and NG patients, these results contribute to an improved understanding of the early impact of PTDM in the kidney glomerulus prior to the development of histologically visible diabetic kidney disease and pave the way towards the identification of novel biomarkers distinguishing PTDM from T2DM with the ultimate goal of developing more effective patient-specific treatments.

### Supplementary Information

**Additional file 1:** **Table S1. **Histological characterization of the three groups: NG, T2DM and PTDM.

**Additional file 2:** **Table S2**. Proteins and antibodies used in immunohistochemistry.

**Additional file 3:** **Table S3. **Number of total, less- and more abundant proteins for each comparison.

**Additional file 4: Table S4. **Number of differentially abundant proteins.

## Data Availability

The mass spectrometry proteomics data have been deposited to the ProteomeXchange Consortium via the PRIDE [20] partner repository with the dataset identifier PXD042188. The clinical data that support the findings of this study are available on request from the corresponding author. Individual, deidentified participant data are not freely available because of the risk of patient reidentification. However, interested parties can request access to deidentified participant data or anonymized clinical study reports through submission of a request for access to the corresponding author, provided that the necessary data protection agency and ethical committee approvals are provided in compliance with relevant legislation.

## Abbreviations

ACN: Acetonitrile
BMI: Body Mass Index
BP: Biological Process
CC: Cellular Component
CKD: Chronic kidney disease
DAB: 3,3'-Diaminobenzidine
DD: Dead donor
DM: Diabetes Mellitus
DKD: Diabetic Kidney Disease
DTT: Dithiothreitol
eGFR: Estimated Glomerular Filtration Rate
ESRD: End-Stage Renal Disease
FA: Formic Acid
FASP: Filter Aided Sample Preparation
FFPE: Formalin-Fixed Paraffin Embedded
GO: Gene Ontology
HbA1C: Glycated haemoglobin
KEGG: Kyoto Encyclopedia of Genes and Genomes
LCM: Laser Caption Microdissection
LD: Living donor
MF: Molecular Function
NG: Normoglycaemia
OGTT: Oral Glucose Tolerance Test
PCA: Principal Component Analysis
pretx: Pretreatment
PPI: Protein‒Protein Interaction
PTDM: Posttransplant Diabetes Mellitus
posttx: Posttreatment
T2DM: Type 2 Diabetes Mellitus
TEM: Leukocyte Transendothelial Migration
TFA: Trifluoroacetic Acid
