# Supplementary material for: Glomerular proteomic profiling reveals early differences between preexisting and de novo type 2 diabetes in human renal allografts
Source: BMC Nephrol. 2023 Aug 25;24:254. doi: 10.1186/s12882-023-03294-z (PMC10464146; doi:10.1186/s12882-023-03294-z)
Supplement: Supplementary file 1 — Additional file 1: Table S1. Histological characterization of the three groups: NG, T2DM and PTDM. [file 12882_2023_3294_MOESM1_ESM.docx]

**TABLE S1. Histological characterization of the three groups: NG, T2DM and PTDM**

| Sample | N° of glomeruli | % sclerotic glomeruli | % of glomeruli with glomerulonephritis | Interstitial fibrosis: ci score | Tubular atrophy: ct score |
| --- | --- | --- | --- | --- | --- |
| NG1 | 9 | 0,33 | 0 | 6-25% | 1-25% |
| NG2 | 11 | 0 | 0 | 0-5% | 1-25% |
| NG3 | 9 | 0 | 0 | 6-25% | 1-25% |
| NG4 | 19 | 0 | 0 | 6-25% | 1-25% |
| Total glomeruli | 48 |  |  |  |  |
| Number Glomeruli/sample | 12 |  |  |  |  |
| Ci0 |  |  |  | 0,25 |  |
| Ci1 |  |  |  | 0,75 |  |
| Ct0 |  |  |  |  | 0 |
| CT1 |  |  |  |  | 1 |
| T2DM1 | 18 | 0 | 0 | 0-5% | 0 |
| T2DM2 | 13 | 0,15 | 0 | 0-5% | 1-25% |
| T2DM3 | 7 | 0,14 | 0 | 0-5% | 1-25% |
| T2DM4 | 21 | 0 | 0 | 0-5% | 1-25% |
| T2DM5 | 18 | 0,06 | 0 | 6-25% | 1-25% |
| T2DM6 | 23 | 0,04 | 0 | 0-5% | 1-25% |
| Total glomeruli | 100 |  |  |  |  |
| Number Glomeruli/sample | 16,67 |  |  |  |  |
| Ci0 |  |  |  | 0,83 |  |
| Ci1 |  |  |  | 0,17 |  |
| Ct0 |  |  |  |  | 0,17 |
| CT1 |  |  |  |  | 0,83 |
| PTDM1 | 15 | 0 | 0 | 0-5% | 1-25% |
| PTDM2 | 11 | 0 | 0,09 | 0-5% | 1-25% |
| PTDM4 | 14 | 0 | 0 | 6-25% | 1-25% |
| PTDM5 | 18 | 0,06 | 0 | 6-25% | 1-25% |
| PTDM6 | 18 | 0 | 0 | 0-5% | 1-25% |
| PTDM7 | 8 | 0,13 | 0 | 0-5% | 0 |
| PTDM8 | 19 | 0,11 | 0 | 0-5% | 1-25% |
| Total glomeruli | 103 |  |  |  |  |
| Number Glomeruli/sample | 14,72 |  |  |  |  |
| Ci0 |  |  |  | 0,71 |  |
| Ci1 |  |  |  | 0,29 |  |
| Ct0 |  |  |  |  | 0,14 |
| CT1 |  |  |  |  | 0,86 |
|  |  |  |  |  |  |

**TABLE S1. Histological characterization of the three groups: NG, T2DM and PTDM**. Total number of glomeruli per sample and percentages of sclerotic glomeruli and glomeruli with glomerulonephritis are listed. Banff classification was used to classify interstitial fibrosis (ci score) and tubular atrophy (ct-score). Interstitial fibrosis was classified using the ci score: Ci0 correlates with 0.5% and ci1 with 6-25% affected tissue. Tubular atrophy was classified with ct score: Ct0 stands for no tubular atrophy and ct1 relates to mild atrophy with 1-25% of the tissue being affected. All samples showed less than 25% interstitial fibrosis or tubular atrophy.
