# Supplementary material for: Glomerular proteomic profiling reveals early differences between preexisting and de novo type 2 diabetes in human renal allografts
Source: BMC Nephrol. 2023 Aug 25;24:254. doi: 10.1186/s12882-023-03294-z (PMC10464146; doi:10.1186/s12882-023-03294-z)
Supplement: Supplementary file 2 — Additional file 2: Table S2. Proteins and antibodies used in immunohistochemistry. [file 12882_2023_3294_MOESM2_ESM.docx]

**TABLE S2**. **Proteins and antibodies used in immunohistochemistry**

| Protein | Protein ID | Antibody | Company | Catalog number | Dilution | Retrieval |
| --- | --- | --- | --- | --- | --- | --- |
| CTNND1 | O60716 | Anti-CTNND1 antibody produced in rabbit | Merck | HPA015955 | 1:50 | pH6 |
| LHPP | QH008 | Anti-LHPP antibody produced in rabbit | Merck | HPA009163 | 1:100 | pH6 |
| MLLT4 | P55196 | Anti-AFDN antibody produced in rabbit | Merck | HPA030212 | 1:500 | pH6 |

**TABLE S2**. **Proteins and antibodies used in immunohistochemistry.** Conditions for antibodies against Catenin Delta 1 (CTNND1), Phospholysine Phosphohistidine Inorganic Pyrophosphate Phosphatase (LHPP) and Afadin (MLLT4) used for immunohistochemical staining in this study, including company and catalog number.
