# Supplementary material for: Glomerular proteomic profiling reveals early differences between preexisting and de novo type 2 diabetes in human renal allografts
Source: BMC Nephrol. 2023 Aug 25;24:254. doi: 10.1186/s12882-023-03294-z (PMC10464146; doi:10.1186/s12882-023-03294-z)
Supplement: Supplementary file 4 — Additional file 4: Table S4. Number of differentially abundant proteins. [file 12882_2023_3294_MOESM4_ESM.docx]

**TABLE S4. Number of differentially abundant proteins**

|  | **Total** | **Up-** | **Down-** |
| --- | --- | --- | --- |
| **PTDM vs. T2DM** | 60 | 27 | 33 |
| **NG vs. PTDM** | 144 | 21 | 123 |
| **NG vs. T2DM** | 126 | 33 | 93 |
